# A Structural Overview of Vascular Endothelial Growth Factors Pharmacological Ligands: From Macromolecules to Designed Peptidomimetics

**DOI:** 10.3390/molecules26226759

**Published:** 2021-11-09

**Authors:** Xiaoqing Ye, Jean-François Gaucher, Michel Vidal, Sylvain Broussy

**Affiliations:** 1 Faculté de Pharmacie de Paris, Université de Paris, CiTCoM, 8038 CNRS, U 1268 INSERM, 75006 Paris, France; xiaoqing.ye@etu.u-paris.fr (X.Y.); michel.vidal@u-paris.fr (M.V.); 2 Laboratoire de Cristallographie et RMN Biologiques, Faculté de Pharmacie de Paris, Université de Paris, CiTCoM, 8038 CNRS, 75006 Paris, France; jean-francois.gaucher@u-paris.fr; 3 Service Biologie du Médicament, Toxicologie, AP-HP, Hôpital Cochin, 75014 Paris, France

**Keywords:** vascular endothelial growth factors, ligands, structures, pharmacological inhibition, macromolecules, peptides

## Abstract

The vascular endothelial growth factor (VEGF) family of cytokines plays a key role in vasculogenesis, angiogenesis, and lymphangiogenesis. VEGF-A is the main member of this family, alongside placental growth factor (PlGF), VEGF-B/C/D in mammals, and VEGF-E/F in other organisms. To study the activities of these growth factors under physiological and pathological conditions, resulting in therapeutic applications in cancer and age-related macular degeneration, blocking ligands have been developed. These have mostly been large biomolecules like antibodies. Ligands with high affinities, at least in the nanomolar range, and accurate structural data from X-ray crystallography and NMR spectroscopy have been described. They constitute the main focus of this overview, which evidences similarities and differences in their binding modes. For VEGF-A ligands, and to a limited extent also for PlGF, a transition is now observed towards developing smaller ligands like nanobodies and peptides. These include unnatural amino acids and chemical modifications for designed and improved properties, such as serum stability and greater affinity. However, this review also highlights the scarcity of such small molecular entities and the striking lack of small organic molecule ligands. It also shows the gap between the rather large array of ligands targeting VEGF-A and the general absence of ligands binding other VEGF members, besides some antibodies. Future developments in these directions are expected in the upcoming years, and the study of these growth factors and their promising therapeutic applications will be welcomed.

## 1. Introduction

### 1.1. Vasculogenesis, Angiogenesis, and Lymphangiogenesis

Vasculogenesis and angiogenesis are two mechanisms of blood vascular networks formation, growth, and remodeling. Vasculogenesis is defined as the process of new blood vessel formation emerging during the embryonic development of the cardiovascular system, which generates the early vascular plexus and subsequently primitive blood vessels [1]. Angiogenesis refers to the process by which new blood vessels take shape from pre-existing ones based on the endothelial cell “sprouting” and intussusceptive microvascular growth [2]. The vascular endothelial growth factor A (VEGF-A) is the main factor responsible for endothelial cell migration, proliferation and tube formation, and other VEGF family members and their receptors play important roles in vasculogenesis and angiogenesis [3,4,5]. Moreover, the VEGF family and their receptors play a role in lymphangiogenesis [6,7,8,9,10]. Blood and lymphatic vessels dysfunction is associated with many pathological conditions such as chronic edema, tumor metastasis, ocular diseases, and impaired immune response [11,12]. Although lymphangiogenesis has been less studied than vasculogenesis and angiogenesis at present, there is no doubt that exploring further the functions and molecular mechanisms of this vascular system must bring some new inspirations for biologists and chemists.

### 1.2. The VEGF Family of Growth Factors

**Figure 1:**
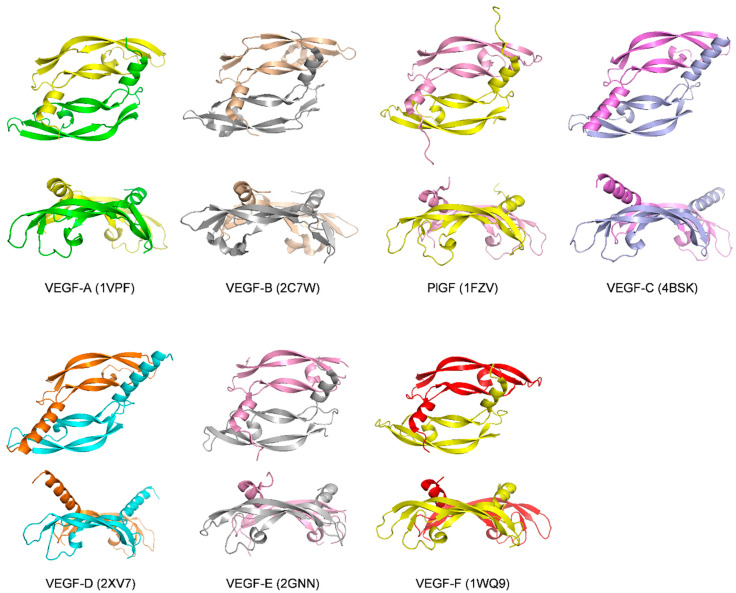
Structures of the receptor-binding domains of VEGF family members. The top representation shows the view along the two-fold symetry axis of VEGF, while the bottom representation shows a perpendicular view. PDB codes are given in parenthesis. For VEGF-C (4BSK), only the growth factor is shown, while the structure includes a receptor fragment.

**Figure 2:**
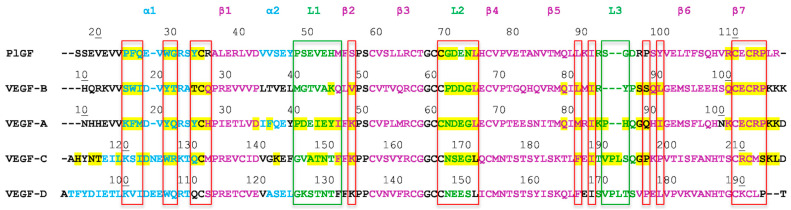
Sequence alignment of the receptor-binding domains of mammalian VEGF subtypes. The secondary structures are reported as annotated by the PDB: note that the second helix α2 was often a single 3 (10) turn. The main residues implied at the interface with domain 2 and domain 3 of the receptors are in red and green boxes, respectively. Most of them are not conserved through the types. VEGFR-specific binding is associated with sequence features: for example, the presence of aromatic residue in position 26 (PlGF numbering; 17 VEGF numbering) is associated with VEGFR-1 binding. Residues observed at the interfaces with their receptor(s) for each type of VEGF are highlighted in yellow.

**Figure 3:**
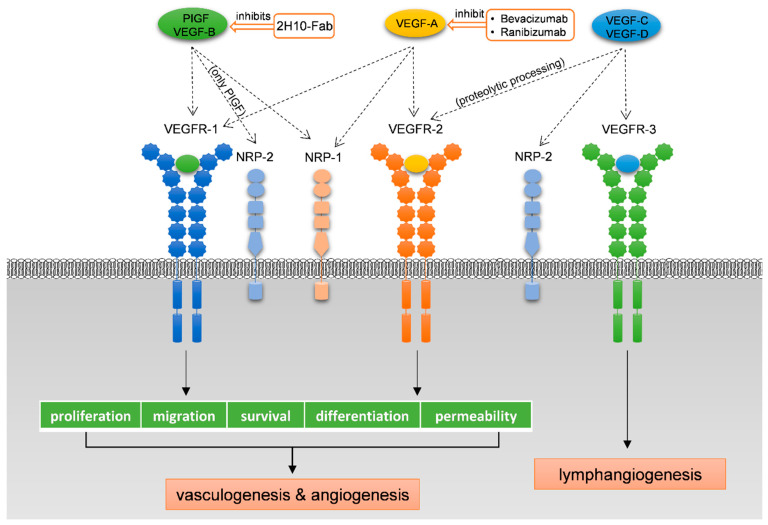
VEGF signaling pathways and some representative antagonists. VEGF-A binds both VEGFR-1 and VEGFR-2, whereas VEGF-B and PlGF only bind to VEGFR-1. VEGFR-1 modulates the action of VEGFR-2 and acts as a decoy or trap for VEGF-A. These pathways are relevant to vasculogenesis and angiogenesis. On the other hand, VEGF-C and VEGF-D bind to VEGFR-3, thereby regulating lymphangiogenesis and VEGFR-2 after proteolytic processing [36]. VEGF-A and VEGF-B can bind to co-receptor NRP-1, which promotes the activation of VEGFRs but is not essential [37,38]. PlGF isoforms (PlGF-2 and PlGF-4) can bind to both NRP-1 and NRP-2 as they have the insert of the heparin-binding domain [39]. NRP-2 binding of VEGF-C/D could lead to the formation of VEGF-C(D)/VEGFR-3/NRP-2 ternary signaling complexes, subsequently facilitating the physiological or pathological lymphangiogenesis [40].

The VEGF family of proteins belongs to the platelet-derived growth factor (PDGF) subgroup of the growth factor cystine knot group [13]. They are characterized by a knotted arrangement of three intramolecular disulfide bridges and the formation of homodimers through two additional disulfide bridges. This VEGF protein family comprises VEGF-A, VEGF-B, VEGF-C, VEGF-D, and placental growth factor (PlGF) in mammals [14]. In addition, VEGF homologs are expressed in Orf viruses, named as VEGF-E, and in snake venom, called VEGF-F [15,16,17,18]. The receptor-binding domains (RBD) of these growth factors, located in the N-terminal part, have been crystallized (Figure 1). Initially designated as “VEGF” in early 1989 by N. Ferrara et al. [19], VEGF-A is the most extensively studied member of the VEGF family. VEGF-A term is used in this review to distinguish it from other family members. Owing to the differences in exon splicing processes (and additional proteolytic processing by plasmin), multiple isoforms of VEGF-A have been identified: VEGF_110_, VEGF_121_, VEGF_145_, VEGF_162_, VEGF_165_ (mVEGF_164_ in mice), VEGF_165b_, VEGF_183_, VEGF_189_, and VEGF_206_ [20,21,22]. VEGF_165_ is the most abundant VEGF-A isoform. The most characteristic feature of these isoforms is their different affinities for heparin, resulting from differences in their C-terminal heparin-binding domains (HBD) [22,23]. After the discovery of VEGF-A, four other homologs have been identified successively, and the timeline is PlGF identified in 1991 [24], VEGF-B and VEGF-C identified in 1996 [25,26], and VEGF-D identified in 1998 [27]. All of them have been less explored than VEGF-A. Alternative splicing of mature mRNAs transcribes two isoforms of VEGF-B and four isoforms of PlGF, which consist of VEGF-B_167_, VEGF-B_186_ and PlGF-1 (PlGF_131_), PlGF-2 (PlGF_152_), PlGF-3 (PlGF_203_), and PlGF-4 (PlGF_224_), respectively [28,29,30,31]. The high degree of sequence homology of some VEGFs allows them to bind to the same receptors: VEGF-B, PlGF, and VEGF-A can target VEGFR-1 and the co-receptor neuropilin-1 (NRP-1) [24,32]. VEGF-C and VEGF-D, which are involved in lymphangiogenesis, bind to VEGFR-2, VEGFR-3, and NRP-2. (Figure 2 and Figure 3, and Section 1.3) [7,33]. Unlike other VEGFs, no splice isoforms of VEGF-C and VEGF-D have been reported to date. Their different forms emanate from proteolytic processing [10]. Biological activities of VEGFs in physiological and pathological conditions are still under investigation and have been reviewed [5,13]. Although VEGFs are usually present as homodimers, natural and biologically active heterodimers have been detected, particularly between VEGF-A and PlGF [34,35].

### 1.3. The VEGF Receptors and Co-Receptors

Vasculogenesis, angiogenesis, or lymphangiogenesis are all tightly regulated by VEGF receptors (VEGFRs), associated with co-receptors neuropilins [33,41]. VEGFs, VEGFRs, and neuropilins, construct a regular and effective cell signaling network (Figure 3) modulating endothelial cells proliferation, migration, and survival. VEGFRs are homodimeric tyrosine kinase receptors structurally related to the PDGF receptor family. The classic feature of VEGFR is having seven immunoglobulin (Ig)-like domains in the extracellular domain (ECD), a single transmembrane helix, and a tyrosine kinase domain in the intracellular portion [42], which can be activated by transphosphorylation upon binding to VEGF. There are three main types of VEGF receptors related to VEGFs signaling; they are VEGF receptor 1 (VEGFR-1, sometimes also referred to as Flt-1), VEGF receptor 2 (VEGFR-2, sometimes also referred to as Flk-1 in mice and KDR in humans), and VEGF receptor 3 (also referred to as Flt-4, involved in lymphangiogenesis), usually expressed in endothelial cells [33]. VEGF-A can bind to VEGFR-1 and VEGFR-2, VEGF-B and PlGF can bind to VEGFR-1, and VEGF-C and VEGF-D can bind to VEGFR-3 and to VEGFR-2 after proteolytic cleavage [43,44]. NRPs have initially been discovered as independent receptors for class 3 semaphorins, a family of soluble molecules with neuronal guidance functions. Remarkably, they are now identified as co-receptors for the VEGFRs [45] and a wide variety of transmembrane receptors. The extracellular portion of NRPs folds into five domains referred to as a1, a2, b1, b2, and c, followed by a transmembrane helix and a short, approximately 40-residues cytoplasmic tail [46]. VEGF-A and VEGF-B both can bind to co-receptor NRP-1, which can promote the activation of VEGFRs but is not indispensable [37]. PlGF-2 and PlGF-4 can bind to both NRP-1 and NRP-2 as they have the insert of the heparin-binding domain [38,39]. In addition, NRP-2 binding of VEGF-C/D could lead to the formation of VEGF-C(D)/VEGFR-3/NRP-2 ternary signaling complexes, subsequently facilitating the physiological or pathological lymphangiogenesis [40,47]. It is generally considered that VEGFs without a heparin-binding domain are incapable of interacting with NRPs or cannot form ternary complexes even though interactions take place [48,49,50].

### 1.4. Scope of the Review

As VEGFs are crucial regulators for blood or lymphatic vessel growth and survival, the dysregulation of these cytokines causes some diseases. It has been demonstrated that the VEGFs play an important role in tumor growth and metastasis, age-related macular degeneration (AMD), diabetic and hypertensive retinopathy [51,52,53,54]. Since then, substantial work has been done to produce agents targeting VEGFs (especially VEGF-A), VEGF receptors, or VEGF-regulated pathways. Some agents have already been approved by U.S. FDA and the European Medicines Agency, such as VEGF-A targeting antibodies bevacizumab and ranibizumab, which now are widely used therapeutics in oncology and eye diseases, respectively [44,55,56]. In addition, developing selective and potent ligands targeting VEGFs can give deeper insights into their mechanisms and functions in physiological or pathological conditions, which are not yet completely understood.

This review will provide an exhaustive and up-to-date overview of reported VEGFs ligands, their co-structures, and their binding affinities [13]. A detailed description of the discovery of VEGFs and their biological activities, and of ligands of VEGFRs is outside the scope of this review. The detailed results of clinical trials involving antibodies, proteins, and aptamers ligands, as well as gene therapy of VEGF-A, are also outside the scope of this review. The reader is referred to recent articles on these topics [36,44,57]. The novelty of the present review is the focus on the binding modes and the structures of ligands with high affinity and specificity for VEGFs, for which robust information is available, i.e., X-ray crystallography or NMR data. This gives a unique structural and ligand-based perspective, opposed to the description of biological effects of ligands in other published review articles. The ligands mostly include domains of proteins like receptor fragments, antibodies, and some peptides. They will be classified according to their nature and molecular size because we believe it is important for their pharmacological properties.

In early 1971, the novel concept that anti-angiogenic therapy could be a potential treatment inhibiting tumorigenesis and tumor metastasis had been proposed [58]. Recent results on the biology of the VEGF family indicate that other pathologies can be targeted. The list of known ligands of VEGFs summarized herein should provide chemists, biologists, and pharmacists an up-to-date picture of the current knowledge, and help develop a variety of new molecular structures of reduced sizes that provide novel pharmaceuticals for biological and clinical studies.

## 2. Ligands of VEGF-A

The structure of the full-length VEGF-A_165_ has not been determined yet. However, the structures of the two fragments have been successfully solved. The three-dimensional structure of the receptor-binding domain (RBD, residues 1–110, named VEGF_110_) has been solved by X-ray crystallography (PDB codes: 1VPF and 2VPF) [59,60] and by NMR (1QTY) [61], which proved that VEGF-A was a member of cystine-knot growth factor superfamily. The structure of the heparin-binding domain (HBD, residues 111–165, named VEGF_55_) has been solved by NMR (PDB codes: 1VGH and 2VGH) [62] and refined further by the same method (PDB code 1KMX) [63].

### 2.1. Receptors and Receptor Fragments

The receptors binding to VEGFs comprise VEGFR-1, VEGFR-2, VEGFR-3, and co-receptors NRP-1 and NRP-2. As mentioned above, VEGF-A can bind to VEGFR-1 and VEGFR-2 but not VEGFR-3. VEGFR-1 has the highest binding affinity for VEGF-A (K_d_ value is ≈10–20 pM), while VEGFR-2 has a lower binding affinity (K_d_ value is ~100–125 pM) [64,65,66]. The extracellular ligand-binding domains of VEGFRs consist of seven Ig-like domains, but the binding site for VEGF-A is mostly located at the second domain of VEGFR-1 (VEGFR-1_D2_) and the second and third domains of VEGFR-2 (VEGFR-2_D2-3_) [67,68].

**Figure 4:**
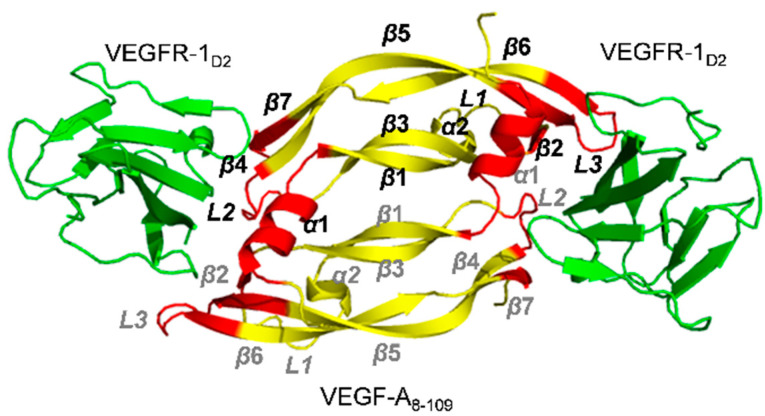
Ribbon representation of the VEGFR-1_D2_ & VEGF-A complex (PDB 1FLT). VEGF-A is colored in yellow, with the binding epitopes in red, and the VEGFR-1_D2_ domains are colored in green. Secondary structures are labeled in black for one monomer of VEGF-A and in grey for the other monomer.

Genentech Inc. disclosed two co-crystal structure of VEGF-A_8-109_ in complex with VEGFR-1_D2_ (Figure 4, PDB code: 1FLT and 1QTY) in 1997 and 1999 [61,69]. The epitopes of VEGF-A in contact with the VEGFR-1_D2_ include residues from one monomer: (1) the N-terminal helix α1 (16–27), (2) the loop 2 connecting β3 to β4 (61–66), (3) the strand β7 (103–106), and residues from the other monomer: (4) the strand β2 (46–48) and (5) strands β5 and β6 together with the connecting loop 3 (79–91). These authors also conducted a domain deletion study of the extracellular domains of VEGFR-1. They showed that the affinity of domain 2 binding to VEGF-A is only 60-fold weaker than that of the full extracellular domain. Moreover, any fragments of VEGFR-1, including domain 2 and domain 3, simultaneously had the same binding affinity as the full ECD [69]. Thus, the second Ig-like domain of VEGFR-1 is necessary and sufficient for high-affinity binding.

**Figure 5:**
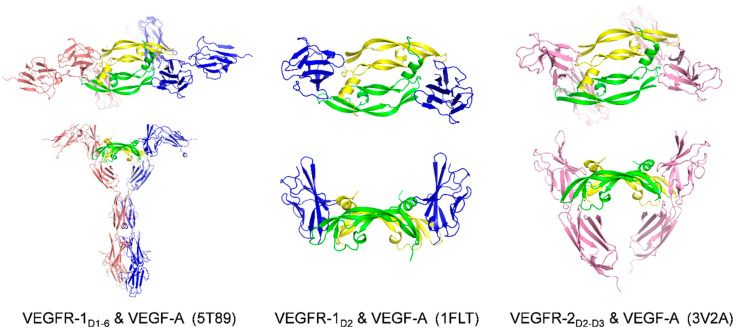
Ribbon representation of the receptor domains/VEGF-A complexes. VEGF-A is colored in green and yellow, and the receptor domains are colored in red and blue. The top representation shows the front view for each complex, while the bottom representation shows the side view. PDB codes are given in parenthesis.

Then in 2017, S. Markovic-Mueller et al. reported the X-ray structure of the full-length VEGFR-1 extracellular domain (D1-D6) in complex with VEGF-A (Figure 5 PDB code: 5T89) [70]. They used a combination of X-ray crystallography, single-particle electron microscopy, and molecular modeling for structure determination and validation. The structure revealed some aspects of the mechanism of ligand-induced receptor dimerization and activation through domain interactions. Still, part of the description of molecular contacts was tentative due to the low resolution (d = 4 Å).

A biochemical assay demonstrated that the binding affinity of VEGFR-2_D1-2_ to VEGF-A is over 1000-fold weaker than that of VEGFR-2_D2-3_ to VEGF-A [71]. The high affinity of VEGFR-2_D2-3_ to VEGF-A was subsequently demonstrated by a thermodynamic and biophysical analysis [72]. Therefore, unlike VEGFR-1, both second and third Ig-like domains of VEGFR-2 are necessary for high-affinity binding. The co-crystal structure of VEGFR-2_D2-3_ binding to the VEGF-A (PDB code: 3V2A) was solved along with co-structure of VEGF-E and VEGFR-2_D2-3_ complex (PDB code: 3V6B) in 2012 by M. S. Brozzo et al. Their work contributed to the structural analysis of VEGFR-2 binding to its major ligands VEGF-A, VEGF-C and VEGF-E [72]. Because VEGF-E is not mammalian and not a prevalent member of the VEGF family, we will not discuss it extensively in this review.

Non-natural protein constructs using VEGFR fragments have been produced to bind VEGF-A with high affinity. The first demonstration was achieved with a chimeric protein constructed by joining the ECD of VEGFR-1 with an IgG. Its capability to block VEGF-induced neovascularization in mice eyes was demonstrated [73]. In another study, the VEGFR-1_D2_ fragment, being sufficient for high-affinity binding, has been fused to the Fc portion of an IgG1 with a glycine linker [74]. Moreover, the oligomerization (dimers and tetramers) of the VEGFR-1_D2_ with short peptides and PEG linkers improved the binding affinity for VEGF-A by up to 200-fold, mainly by slowing down the dissociation rate [75]. One of such chimeric molecules is aflibercept, which is currently used in the clinic to treat AMD. It is constituted of VEGFR-1_D2_ and VEGFR-2_D3_ fused to human Fc region of IgG1 (MW = 96.6 kDa) [76].

**Figure 6:**
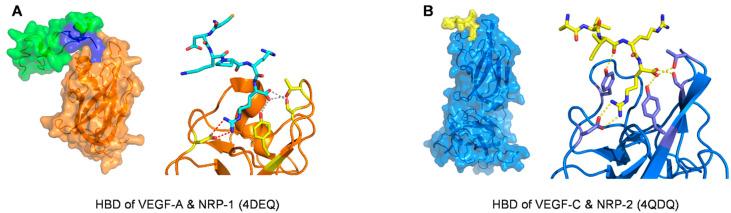
(**A**). Structure of the HBD of VEGF-A (exon 7 in green and exon 8 in blue) in complex with neuropilin-1 (orange) (**B**). Structure of the C-terminal residues of the HBD of VEGF-C (yellow) in complex with neuropilin-2 (marine). Hydrogen bonds are indicated by dashed lines. PDB codes are given in parenthesis.

NRP-1 is identified as a co-receptor of VEGF-A, usually collaborating with VEGFRs and facilitating their efficient ligand binding. M. W. Parker et al. reported the first detailed picture of the structural basis for the binding of NRP-1 and VEGF-A. The binding interface involved regions encoded by both exons 7 and 8 of VEGF-A. Exon 7 encoded residues primarily govern selectivity, whereas exon 8 encoded residues mainly mediate high-affinity binding [77]. A fusion of human NRP-1-b1 (residues 274–429) covalently linked to the VEGF-A HBD (115–165) was constructed for crystallization. Human and mouse HBD sequences differ by only a single residue in their N terminus, and mouse residue numbering was used in this structural study (PDB code: 4DEQ, Figure 6).

### 2.2. Antibodies and Antibody Fragments

Several antibodies and antibody fragments have been developed to bind VEGF-A. The following section will list these ligands in decreasing size order, from the full antibodies (size ≈ 150–160 kDa) to Fab fragments (Fabs, the antigen-binding fragment, size ≈ 55 kDa), single-chain variable fragments (scFvs, size ≈ 30 kDa), and finally single-domain antibodies (sdAbs, which are also called nanobodies and abbreviated Nbs, size ≈ 12–14 kDa).

**Figure 7:**
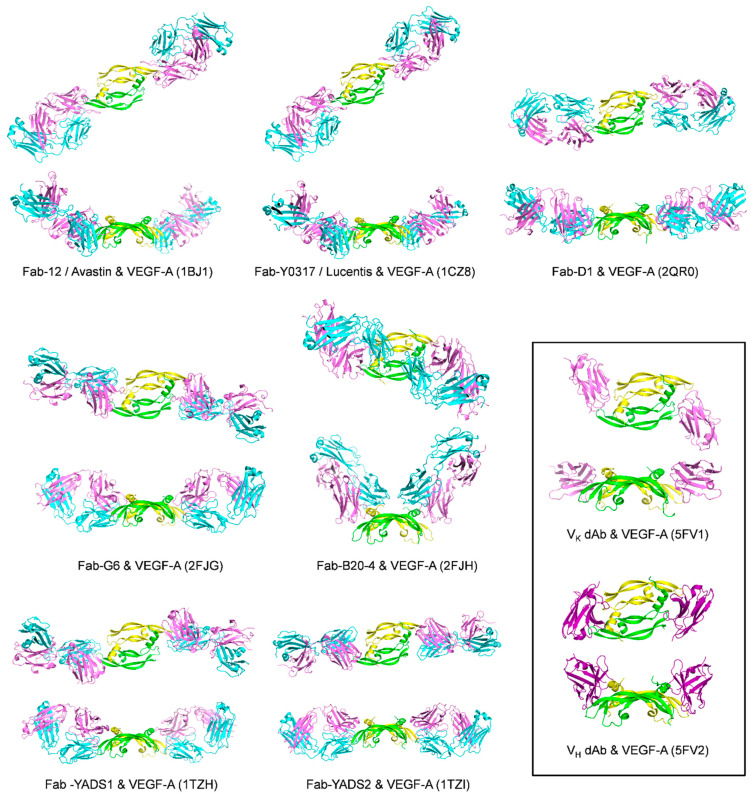
Ribbon representation of antibody/VEGF-A complexes. VEGF-A is colored in green and yellow, and the antibodies are colored in various shades of pink and blue. The top representation shows the front view for each complex, while the bottom representation shows the side view. The insert shows the domain antibody (dAb)/VEGF-A complexes. PDB codes are given in parenthesis.

The first demonstration of inhibiting tumor growth by targeting VEGF-A in vivo was achieved with the murine anti-human VEGF-A monoclonal antibody A.4.6.1, in nude mice transplanted with human tumors [78]. This antibody was subsequently humanized and used in the clinic as bevacizumab (Avastin) to treat cancer and later AMD [44,56]. A crystal structure of VEGF-A in complex with the Fab-12 portion of the antibody has been solved (PDB code: 1BJ1) [79]. It binds VEGF-A with high affinity, mainly to the β5-β6 epitope (Figure 7 and Table 1). The binding characteristics and in vitro activities of bevacizumab and other molecules used in the clinic, ranibizumab (see below) and aflibercept, have been extensively studied and compared [80].

Further improvements of the Fab 12 portion through complementarity-determining region (CDR) mutations followed by affinity selection using monovalent phage display gave the Fab variant Y0317. This final variant incorporated six mutations and had a more than 100-fold higher affinity for VEGF-A than Fab 12. A crystal structure of VEGF-A and this Y0317 Fab demonstrated that they share similar binding epitopes on VEGF-A and evidenced good correlations between improvements in binding affinity and improved hydrogen bonding and Van der Waals interaction with the growth factor (PDB code: 1CZ8) [83]. The Fab variant Y0317 was called ranibizumab (Lucentis). It is human-specific and is used in the clinic for the treatment of cancer and AMD. A structure of a new bevacizumab Fab mutant in complex with VEGF-A has been deposited in the PDB (accession code 6BFT) but has not been published yet.

Genentech Inc. subsequently developed anti-human VEGF-A Fabs by phage display with restricted amino acid diversity in the CDR region. High-affinity Fab ligands were identified (K_d_ ≈ 2–10 nM) with only four amino acids possibilities (tyrosine, alanine, aspartate, serine). The residue tyrosine was overwhelmingly present at the binding interface. Crystal structures of two complexes were solved (VEGF-A and antibody YADS1 Fab, PDB code: 1TZH [88] and VEGF-A and antibody YADS2 Fab, PDB code: 1TZI), showing that the binding epitope on VEGF-A was similar to the previously described Fabs (see above and Table 1) [88]. Moreover, in another study with synthetic phage libraries, a large diversity of high-affinity anti-VEGF-A Fabs has been discovered. The process started with Fabs identified with a minimalist binary library (only serine and threonine), further optimized through a gradual increase in amino acid diversity. One of the best ligands, Fab-D1, has been co-crystallized with VEGF-A (PDB code: 2QR0, Figure 7 and Table 1) [89].

Phage-displayed synthetic Fab libraries with a single framework scaffold and variability in the CDR identified new high-affinity antibodies targeting murine VEGF-A [86]. Improvements of this synthetic antibody phage-display library led to discovering two additional antibodies named G6 and B20 (studied both as Fabs or full IgGs) and their variants. In contrast with bevacizumab, these new antibodies can bind both murine and human VEGF-A with high affinities [100]. Their binding epitopes on VEGF-A were studied by alanine-scanning mutagenesis and structural analysis of complexes with VEGF-A (for G6-Fab PDB code: 2FJG, and for B20-4 Fab, PDB code: 2FJH, Figure 7) [87]. Whereas bevacizumab and all Fab variants described above bind to the β5-β6 epitope of VEGF-A, partly overlapping with the receptor binding site, G6 and B20-4 bind in a manner that matches more closely to the receptor-binding epitopes (Figure 7) [87].

Antibody mimetics called Fab-PEG-Fab, constituted of Fab portions of ranibizumab or bevacizumab dimerized with a PEG linker, have been synthesized. SPR and ITC measurements demonstrated similar binding affinities for VEGF as the full antibody bevacizumab (K_d_ values in the single-digit nanomolar range). However, ranibizumab’s dimerization changed its binding thermodynamic parameters, the dimer having a largely favorable enthalpy and a largely unfavorable entropy [101].

Recently, dual targeting antibody Fab fragments (DutaFabs) have been developed by Roche Inc. Two separate fragments of the CDR of one human Fv region bind specifically to two distinct targets: VEGF-A and PDGF-BB (human PDGF consituted of two B subunits) [84]. Interestingly, the two targets can be bound simultaneously and with high affinity (Table 1). Crystal structures of the complexes have been described (PDB code: 6T9D for the DutaFab-VEGF-A complex).

GlaxoSmithKline pic has developed a new antibody architecture named VEGF dual-domain antibody (dAb). It is composed of two sets of two distinct domain antibodies (named V_K_ and V_H_) attached to a human IgG1 Fc domain with linkers. The increased binding stoichiometry of four binding sites per dual dAb molecule can explain the observed increase in binding affinity for VEGF-A compared to the monoclonal antibody bevacizumab and the antibody fragment ranibizumab. Each dAb has been co-crystallized with VEGF-A independently: V_K_·dAb, PDB code: 5FV1 and V_H_·dAb, PDB code: 5FV2 (Figure 7). Taking these structures into account and using SEC-MALS analysis, a model of VEGF-A binding by a dual dAb was also proposed, in which a single, dual dAb could sequester two VEGF-A homodimers [85]. Studies in rabbits and non-human primates demonstrated the efficacy of a single injection of a formulation of this dual dAb in a model of wet AMD for 6 months [102].

Single chain variable fragments (scFvs) in which only the heavy chain V_H_ was varied and fixed to a light chain V_L_ (kept constant) via a linker were produced by phage-display and selected to bind VEGF-A with affinities of approximately 100 nM [103]. These scFvs were subsequently converted to the larger Fab format described above and constituted starting points to identify smaller entities made of a single domain, the nanobodies (see below). Recently, the first scFv to come to market, brolucizumab (marketed as Beovu), had been approved for the treatment of neovascular AMD in the US in October 2019 and in the EU in February 2020. It is a humanized monoclonal anti-VEGF-A scFv produced in *Escherichia coli* cells by recombinant DNA technology. Notably, brolucizumab is effective with injections in the eyes every 3 months [104,105,106].

Nanobodies targeting VEGF-A and, more generally, proteins involved in angiogenesis were reviewed in 2017 [107]. Compared to full antibodies, the small size of nanobodies is generally considered an advantage, conferring better properties previously detailed [107]. For example, nanobodies with affinity values for the receptor-binding site of VEGF in the low nanomolar range have been identified, and their high stability has been demonstrated [108]. Oligoclonal nanobodies have also been produced and have shown a synergistic inhibition of VEGF-induced proliferation and tube formation in HUVEC that was more efficient than any individual nanobody [109]. In a recent study, a 25-residues peptide (mimotope) derived from the CDR3 region of a VEGF binding nanobody was designed and was shown to bind VEGF with a similar affinity as the parent nanobody [110]. To our knowledge, only structural data produced by molecular modeling was published regarding the interaction between VEGF-A and nanobodies.

### 2.3. Aptamers

Many aptamers have been developed to target VEGF-A, and some have been optimized to reach extremely high affinities. Anti-VEGF DNA-based aptamers have been recently extensively reviewed [57]. Therefore, we will only briefly summarize some key aspects regarding affinity and specificity and the scarce structural information. Pegaptanib is a pegylated RNA aptamer able to interact with the heparin-binding domain of VEGF-A with picomolar activity [111]. It was one of the first aptamers targeting VEGF-A to be developed. It has been used in the clinic (as Macugen) to treat AMD before its replacement by the more efficient bevacizumab and aflibercept in 2011 [36]. Most aptamers target the HBD, and few target the RBD. Affinities for VEGF-A in the picomolar range have been often obtained, mostly determined by SPR. In particular, very high affinities were obtained with the use of unnatural DNA bases (with the ExSELEX process) and multimeric constructs (dimers and trimers). Anti-VEGF-A DNA-based aptamers have been used for drug delivery, protein-affinity purification, and for in vitro detection of VEGF-A [57]. A literature search revealed the absence of high-resolution structural data for the complexes formed between aptamers and VEGF-A.

### 2.4. Small Proteins and Peptides

A few small proteins (of 50–60 amino acids) and peptides (of 15–20 amino acids) have been identified, mostly by phage-display technology. Their structures are not related to receptor or antibody fragments, but their binding sites on VEGF-A overlap with the receptor-binding site. Small proteins and peptides of molecular weights between 500 and 5000 Da occupy a chemical space between small molecules and larger proteins. Peptides are less structured than larger proteins in solution. This leads to a low proportion of the bioactive conformation(s) in the conformational ensemble, and therefore to a generally lower affinity than larger proteins due to an entropic penalty of binding. To avoid this drawback, a cyclization strategy is often chosen to design bioactive peptides by phage-display and chemical synthesis. It results in improved conformational stability and proteolytic resistance [112].

**Figure 8:**
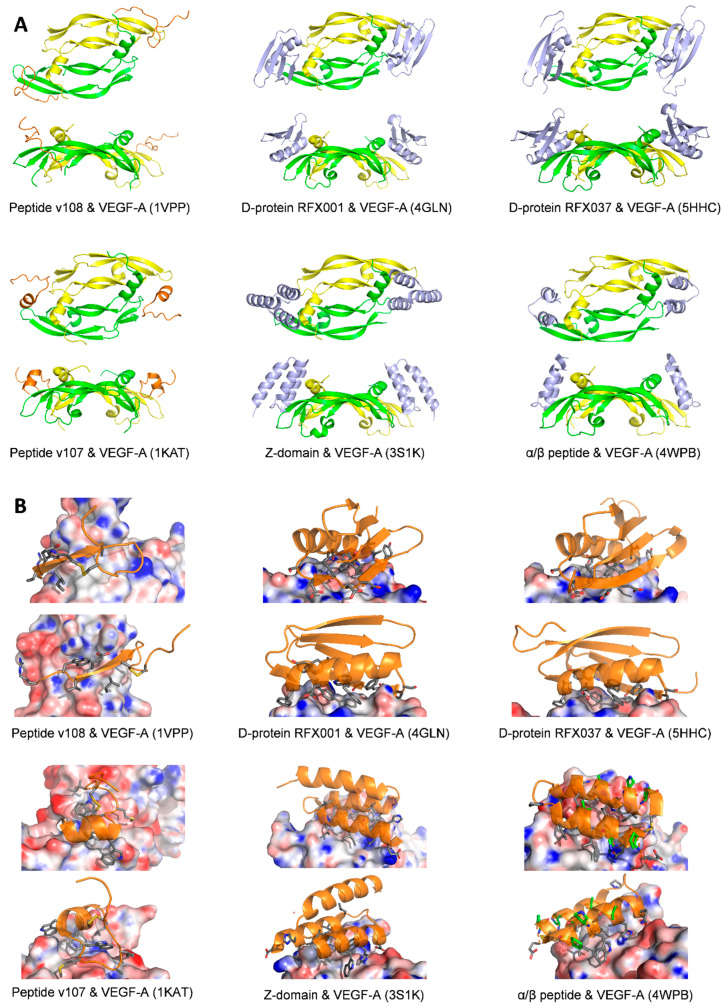
(**A**). Ribbon representation of peptide/VEGF-A complexes. VEGF-A is colored in green and yellow, and the peptides are colored in various shades of orange and blue. The top representation shows the front view for each complex, while the bottom representation shows the side view. PDB codes are given in parenthesis. (**B**) Details of the interaction of peptides or small protein ligands on the electrostatic surface of VEGF-A, calculated using the Adaptive Poisson-Boltzmann Solver [127]. The side chains of the ligands involved in molecular contacts are in gray sticks and the ribbons in orange. For each complex, two representations show the main interactions. PDB codes are given in parenthesis. For the α/β peptide, the non-natural residues are shown in green.

In an example of improved phage display technology, the method of “mirror image phage display” was used, starting from the scaffold of the B1 domain of streptococcal protein G (56 amino acids), against the enantiomer of natural VEGF-A (*D*-VEGF-A, which was produced by total chemical synthesis with *D*-amino acids). It allowed the identification of a small protein-ligand of *D*-VEGF-A with a K_d_ value of approximately 90 nM. In turn, the total chemical synthesis of this small protein with *D* amino acids gave ligands of natural (*L*-) VEGF-A with the same affinity. This *D*-protein antagonist (D-RFX001) was co-crystallized with VEGF-A by a racemic crystallization process, giving heterochiral complexes solved in two space groups: PDB code 4GLN and 4GLS [92]. The contact surface of D-RFX001 encompasses approximatively 800 Å^2^ which competes for the binding of VEGF with VEGFR-1_D2_. It is dominated by a central aromatic cluster surrounded by polar contacts. Further improvement regarding thermal stability, pharmacokinetic properties, and affinity gave a new D ligand RFX037.D (Table 1), which was also co-crystallized with VEGF-A, PDB code: 5HHD and 5HHC. These are also racemic complexes solved in two different space groups (Figure 8A,B). The low immunogenicity of this *D*-small protein was demonstrated in mice [93].

Ligands of VEGF-A based on the scaffolds of the three-helix 58-residues Z-domain of staphylococcal protein A were identified by phage display by Genentech Inc. Among the Z-domain clones, Z-3B (59 residues) was the best ligand with a K_d_ value of 55 nM. Another reengineered version named mini-Z, of 34 amino acids structured in two helices stabilized by a disulfide constraint, bound VEGF-A with a K_d_ of 38 nM. Two crystal structures of complexes of these ligands with VEGF-A were reported (PDB for Z-3B: 3S1K, and mini-Z: 3S1B). Both Z-domain and mini-Z peptides bind to VEGF at the receptor-binding site. The Z-domain buries 744 Å^2^ of the surface area, mainly composed of van der Waals interactions of aliphatic and aromatic side chains, with five hydrogen bonds and two additional salt-bridges at the site periphery. Imaging in mice xenograft models of colorectal and ovarian cancer with a Z-3B radiolabeled with ^18^F gave, in 2 h, images of comparable quality to those obtained with ^89^Zr-radiolabeled B20 antibody 72 h post-injection. This demonstrated the better tumor penetration capacity of the small protein compared to the antibody [94].

Additional studies based on the Z-domain scaffold described above, cyclized by a disulfide bond, were reported. Incorporation of non-natural amino acids to stabilize helices, in particular β-amino acids and 2-aminoisobutyric acid, resulted in ligands with strongly increased stability towards proteases, without significant loss of affinity for VEGF-A. A crystal structure of an α/β peptide in complex with VEGF-A was reported (PDB code: 4WPB, Figure 8A,B) [95,113]. The non-natural amino acids are located at sites that do not contact the VEGF-A surface. Therefore, the interaction surface is very close to that of the Z-domain.

DARPins (designed ankyrin repeat proteins) are genetically engineered antibody mimetics designed to bind protein targets. Abicipar-Pegol, developed by Molecular Partners, Inc., Zurich-Schlieren, Switzerland, is a DARPin constituted of a 14 kDa recombinant protein (4 ankyrin repeats) with a very high affinity for VEGF-A, linked to a 20 kDa PEG tail [114,115]. Three-phase 3 clinical trials have been conducted, but the FDA rejected it in 2020 because of its higher rate of intraocular inflammation than ranibizumab.

Screening of VEGF-A binding peptides by phage-display by Genentech Inc. resulted in identifying three classes of peptide ligands [116]. All of them bound VEGF-A to overlapping epitopes and were able to inhibit receptor binding. These 19 or 20-mer peptides have been extensively studied by NMR and X-ray diffraction [117]. Among them, the peptide v108 was co-crystallized with VEGF-A_8-109_. (Peptide v108 & VEGF_8-109_, PDB code: 1VPP, Figure 8A,B) [90]. NMR showed that peptide v108 folds in the presence of VEGF. Its central region of nine amino acids forms a type I β-hairpin loop stabilized by an intramolecular disulfide bridge. The N-terminal half folds in β-strand conformation, which binds to the main chain β6 of VEGF. The C-terminal is flexible and does not interact with VEGF. Overall, it resembles the binding mode of the Fab 12 antibody. However, the extended conformation and the scarcity of specific side-chain interactions led the authors to consider the peptide as not being a promising lead for the development of small molecules. 

The structure of peptide v107 in complex with VEGF-A was solved by NMR (PDB code: 1KAT) [91,118]. The peptide folds in the presence of VEGF-A with a C-terminal α-helix and a central β-turn. It wraps around an aromatic core and is stabilized by a disulfide bridge (Figure 8A,B). Although the folding process results in a thermodynamically unfavorable entropy variation [119], the peptide v107 was considered a suitable starting point for further optimization because of its high affinity for VEGF-A, its structuration upon binding, and because it is binding with the growth factor was mainly mediated by specific hydrophobic interactions with its side chains. Additional studies on this v107 peptide include a study in the gas phase of D-amino acids variants [120], the modification with an electrophile chemical function for covalent labeling of VEGF-A [121], the radiolabeling for imaging [122,123], the extension with peptide fragments to improve its affinity as a capture agent [122], the incorporation of non-natural beta-amino acids for increased proteolytic stability (structure of peptide HH4, PDB 6D3O, to be published) [119,124], the shortening of the sequence to 15 amino acids [125], and the stabilization of the α-helix C-terminal end by cyclization for increased affinity (PDB 6Z13, 6ZCD, 6Z3F, 6ZBR, to be published). A bacterial display screening independently identified peptides that were very similar to v107, with comparable affinities for VEGF-A and the same core sequence WE/DWE/D [126].

A cyclic hexapeptide incorporating the Trp/Glu motif found in v107 displayed weak affinity (low millimolar range) for VEGF-A. Still, the demonstration of its specific binding at the receptor-binding site was achieved by NMR spectroscopy [128].

### 2.5. Small Molecules

To the best of our knowledge, there is no small molecule co-crystallized with VEGF-A. The large hydrophobic and flat interaction surface on receptors constitutes a difficult target for small molecules. Only one study reported attempts to find small molecules as VEGF-A ligands [128]. The authors screened libraries of dipeptides, peptides incorporating unnatural amino acids, and small molecules but did not identify ligands of VEGF-A. Molecular modeling indicated that the most probable binding sites for small molecules on VEGF-A are located outside the receptor-binding interface, in clefts between the two monomers of the growth factor. However, screening by NMR of natural products identified a flavonoid scaffold able to bind VEGF-A at the receptor-binding interface, although with weak affinity (K_d_ values are in the low millimolar range).

### 2.6. Analysis of the Binding Modes of VEGF-A Ligands

**Figure 9:**
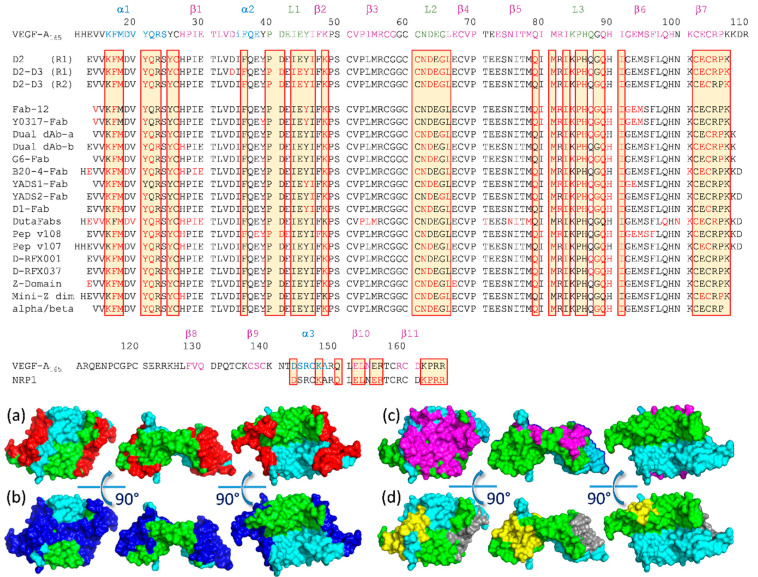
Binding sites of artificial ligands on VEGF-A overlap with the binding sites of VEGF-R. Top: Comparison of the VEGF-A residues buried at the interface with VEGFR-1_D2_ or VEGFR-2_D2D3_ (yellow boxes) and the residues buried at the interface with the available ligands whose co-structures have been solved (in red letterings). Uppercase letters indicate solvent-accessible residues in the VEGF structure. Bottom: representation of the accessible surface of VEGF-A buried at the ligand interface, as identified with PISA [129]. (**a**) Red: VEGFRs interface. (**b**) Blue: ligands interfaces, except v108, v107, and DutaFab. (**c**) Magenta: DutaFab interface. (**d**) The residues buried at v108 or v107 interfaces are in yellow and grey, respectively. PDB-ids are indicated in Table 1.

The VEGFRs domains D2 and D3 bind VEGF-A on several discontinuous epitopes constituting the binding site: the helix α1, the loops L1, L2, and L3, and the strand β7 (Figure 9). All the other known ligands target at least one of these epitopes, thereby inhibiting the growth factor/receptor interaction. The residues of VEGF-A buried at the interface are colored in red in Figure 9. In the literature, we did not find any ligand able to block the interaction by steric hindrance while targeting epitopes outside the binding site.

As listed above, the ligands are diverse in sizes and shapes, and their secondary structures are not shared, even at the binding site. However, some specific non-covalent interactions are shared for each epitope across several ligands. For example, ligands targeting the helix α1 make use of π stacking interactions between their aromatic residues and F17, Y21, and Y25 of VEGF-A. Polar interactions are often present with residues of loop L2, hydrogen bonds with N62, and charge/charge with D63.

## 3. Ligands of PlGF and VEGF-B

Both PlGF and VEGF-B are VEGFR-1 specific ligands and less studied than VEGF-A. Therefore, we will discuss them in the same chapter. The crystal structures of PlGF (PDB code: 1FZV) and VEGF-B (PDB code: 2C7W) were solved by X-ray crystallography in 2001 and 2006, respectively [32,130]. Despite their functional diversities, they showed a highly similar topological structure with VEGF-A (Figure 1) [60].

### 3.1. Receptors and Receptor Fragments

**Figure 10:**
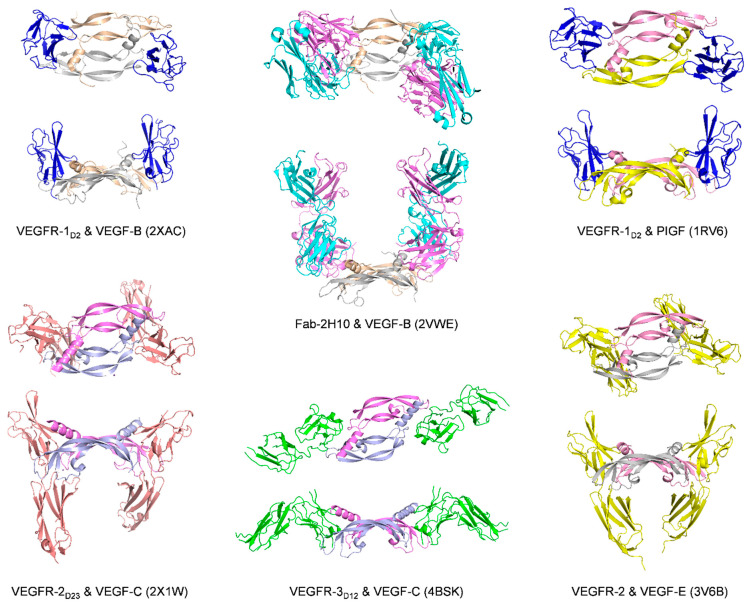
Ribbon representation of ligand/VEGF or PlGF complexes. The top representation shows the front view for each complex, while the bottom representation shows the side view. PDB codes are given in parenthesis.

The co-crystal structure of PlGF and VEGFR-1_D2_ (PDB code: 1RV6, Figure 10) was determined by H.W. Christinger et al. using X-ray crystallography in 2004 [81]. Meanwhile, their research group carried out an SPR competitive binding assay showing that the presence of VEGFR-1_D3_ has a much greater effect on the binding affinity of PlGF than of VEGF-A. This result strongly indicates that the third Ig-like domain of the VEGFRs plays an important part in the specificity of receptor recognition [81]. Then, in 2010, the crystal structure of VEGF-B in complex with VEGFR-1_D2_ (PDB code: 2XAC, Figure 10) was disclosed [96], of which the topological structure is essentially similar to that seen in two complexes of this receptor with VEGF-A and PlGF [69,81]. VEGF-B did not require the VEGFR-1_D3_ for high-affinity binding [131]. Comparing the three co-crystal structures of VEGFR-1D2 with VEGF-A, VEGF-B, and PlGF provides abundant information on how subtle differences influence receptor recognition. Interestingly, the electrostatic surface potentials of the growths factor seem to be complementary to the corresponding surface potential of their cognate receptors [96]. Given that VEGF-A, VEGF-B, and PlGF share the same overall binding mode on VEGFR-1 and some sequence homology, it is intriguing but difficult to explain how receptor recognition and specificity happen and translate into distinct biological activities. In such an attempt, the importance of the L1 loop of the growth factors and its interaction with VEGFR-1_D3_ has been demonstrated [131].

### 3.2. Antibodies and Antibody Fragments

Inhibition of PlGF activity by antibodies has been the subject of several studies since an initial report demonstrated that αPlGF (clone 5D11D4, a mouse PlGF-2 specific blocking antibody) was able to inhibit the growth of VEGF(R)-inhibitor-resistant tumors in mice [132]. A subsequent study with the same antibody confirmed these results and demonstrated additional beneficial activity by inhibiting angiogenesis in eye diseases [133]. The IC_50_ value for inhibition of the mPlGF/VEGFR-1 interaction by the full 5D11D4 antibody was 27 pM and in the low nanomolar range with Fab fragments thereof [133]. However, the broad utility of specific PlGF inhibition (or in combination with VEGF-A inhibition) with antibodies in cancer has been challenged, in particular with the observation that only tumors expressing VEGFR-1 are potential targets [134,135]. In addition, the importance of targeting also the PlGF/NPR-1 interaction to inhibit the growth efficiently and spread of brain tumors has been demonstrated. The authors used an antibody blocking only the PlGF/VEGFR-1 interaction (C9.V2) versus antibodies blocking both interactions (TB-403 and 5D11D4) [136]. Therefore, although it is clear that all the antibodies tested bind PlGF with high affinity and selectivity, additional knowledge regarding the precise binding sites and binding modes may help in understanding their different biological activities. To our knowledge, no experimental structural information (X-ray or NMR) regarding the binding modes of anti-PlGF antibodies has been disclosed. The role of PlGF in antitumor immune response and existing anti-PlGF targeting strategies have been recently reviewed [137]. The humanized anti (human) PlGF antibody TB-403 (RO5323441) has been tested in a few clinical trials, initially a phase I clinical trials in patients with advanced solid tumors [138] and more recently in pediatric subjects with relapsed or refractory medulloblastoma, for which the results will be released in 2022 (https://clinicaltrials.gov/ct2/show/NCT02748135, accessed 29 September 2021).

PlGF-specific nanobodies with affinities in the nM range have been identified by phage-display and studied as monomers and dimers [139,140]. Their anti-angiogenic activity was demonstrated with several in vitro angiogenic assays.

Neutralizing monoclonal antibodies targeting VEGF-B specifically (over VEGF-A) and with high affinity and preventing VEGFR-1 binding have been developed [141]. A crystal structure of the complex between VEGF-B and a neutralizing antibody Fab fragment (Fab-2H10) has been described (PDB code: 2VWE) Fab (133). It displays a unique U-shape topology not previously observed in the VEGF family. This antibody binds VEGF-B at its VEGFR-1 binding site, thereby blocking the interaction between the growth factor and the receptor (Figure 10). The 2H10 antibody (by intravitreal injection) and an scFv fragment thereof (by combined topical and sub-conjunctival injection) have been shown to induce regression of pre-existing blood vessels at the back of the eye in mouse and rats models of eye diseases [142,143]. The antibody 2H10 and the scFv have high affinities and selectivities for human, rat, and mouse VEGF-B, which is convenient for preclinical studies [144].

### 3.3. Small Proteins and Peptides

Using phage display, Pfizer Inc. identified 15-residues peptides binding PlGF-1. Although the affinity of these initial peptides was modest (IC_50_ ≈ 1 µM), extreme improvement in affinity (10^3^) was demonstrated by dimerization through the covalent binding on a CovX antibody scaffold. The antibody scaffold alone did not bind the growth factor and was only used as a template linker. Therefore, the improvement was explained by possible avidity effects. Additional modifications with the incorporation of non-natural amino acids and optimization of the linker position led to a ligand with a K_d_ of 0.1 nM for PlGF-1 and increased stability in serum. Moreover, some specificity of the ligands for human PlGF-1 over human PlGF-2, mouse PlGF, and VEGF-A was demonstrated. In the absence of structural information, the authors propose that the specificity may be explained by binding the peptide to an epitope of the RBD accessible only in PlGF-1, which does not possess an HBD and is hindered by the HBD in other growth factors that possess one [145].

## 4. Ligands of VEGF-C and VEGF-D

The same chapter will discuss VEGF-C and VEGF-D because they share high homology of structure and bind to the same receptors VEGFR-2, VEGFR-3, and NRP-2. They were initially described as having very similar functions. However, recent studies demonstrate that they actually have different binding and regulatory mechanisms in lymphangiogenesis [146]. A crystal structure of VEGF-D alone has been described (PDB code: 2XV7, Figure 1) [147]; however, the crystal structures of VEGF-C are all in complex with fragments of the receptors (see below). A characteristic feature of VEGF-C and VEGF-D compared to other VEGFs is the extended N-terminal α-helix [147]. In VEGF-D (and not VEGF-C), the length of this N-terminal α-helix is critical for VEGFR-2 and VEGFR-3 binding [146].

### 4.1. Receptors and Receptor Fragments

Crystal structures of VEGF-C with fragments of each of its three receptors have been published. In the complex with VEGFR-2_D2-3_ (Domains 2 and 3 of VEGFR-2, PDB code: 2 × 1X and 2 × 1W), the binding epitopes of both proteins were identified, as well as critical residues of the receptor explaining its affinity for VEGF-A and -C (Table 1 and Figure 10) [82]. In the complex between VEGF-C and VEGFR-3_D1-2_ (Domains 1 and 2 of VEGFR-3, PDB code: 4BSK, Figure 10), despite the low resolution and the impossibility to define specific interactions, the binding interface on domain 2 was confirmed, with conserved structural features compared to other members of the VEGF family [98]. Therefore, the presence of domain 3 did not significantly change the dissociation constant of VEGF-C for the receptor fragments: K_d_ for VEGFR-3_D1-2_ was 250 nM, and K_d_ for VEGFR-3D1-3 was 140 nM. However, the K_d_ for VEGFR-3_D1-5_ was 3.7 nM because domain 5 dimerized in the presence of VEGF-C, thereby increasing the total affinity. In addition to the RBD, a complex of the HBD of VEGF-C in interaction with NRP-2 has been solved (PDB code: 4QDQ, Figure 6B, from a fusion construct between NRP-2 and the C-terminal amino acids of the HBD of VEGF-C). The structure involves C-terminal residues of the HBD, terminated by two arginine residues in the sequence, with one of them deep into the binding cleft of the b1 subunit. Therefore, the structure demonstrates the importance of the C-terminal proteolytic cleavage of VEGF-C and the shared mode of binding with the VEGF-A/NRP-1 structure [99]. To our knowledge, there are no co-structures of VEGF-D with receptor fragments.

Soluble forms of VEGFR-3 with high affinities for VEGF-C and -D have been used to explore the effect of blocking this pathway on lymphangiogenesis [148]. Recent reports indicate the emerging roles of VEGF-D in several human diseases [148]. A first-in-class drug candidate, the soluble receptor fragment of VEGFR-3 named OPT-302 (VEGFR-3_D1-3_ fused to the Fc fragment of IgG1), is explored in clinical trials to target VEGF-C and VEGF-D, as monotherapy or combined with VEGF-A blockade, in eye diseases such as neovascular AMD [149].

### 4.2. Antibodies

Several antibodies binding VEGF-C are commercially available [150]. Some of them can block its activity, like VGX-100, which precipitates all forms of human and mouse VEGF-C. The biological activities of VGX-100 on cancer and ocular diseases have been demonstrated in preclinical models. It has been tested in phase I clinical trial (in combination with bevacizumab) for solid tumors [151,152,153]. However, to our knowledge, there is no specific information regarding the binding epitope or the structures of antibodies in complex with VEGF-C.

In a published study, four antibodies binding VEGF-D have been developed (VD1-4, K_d_ ≈ 30–61 nM), VD1 being selective for VEGF-D (in particular over VEGF-C) and able to prevent its binding to the VEGFR-2 and -3 [154]. Additional antibodies able to block the interaction of the growth factor with the receptors are now commercially available, like mAB286, and used to study the biological activities of VEGF-D [146]. Although no co-crystal structures have been published, the binding epitope has been accurately identified for VD1 (in the L2 loop), which was explored as a potential therapeutic antibody [155].

## 5. Ligands of Non-Mammalian VEGFs

Besides the five mammalian VEGFs mentioned above, the VEGF protein family also comprises two non-mammalian VEGFs: VEGF-E and VEGF-F. The research of natural or synthetic ligands is largely unexplored. VEGF-E is an Orf viral homolog of VEGF-A, which contributes to wound re-vascularization and re-epithelialization [156]. Based on sequence similarity technique, the gene encoding a VEGF homolog was discovered in the genome of the Orf virus in 1994 [17]. Orf virus is the type species of the *Parapoxvirus* genus of the *Poxviridae* family, which primarily causes acute pustular skin lesions in sheep and goats and can infect humans [157]. The functional activity of VEGF-E was investigated, and it was shown to mediate angiogenesis via signaling through VEGFR-2 (KDR) but not VEGFR-1 [158]. VEGF-E only shows 25–35% amino acid identity to VEGF-A, although it has a comparable affinity for VEGFR-2 [159]. Despite the lack of a heparin-binding domain, some variants of VEGF-E retain the binding to neuropilin-1 [160]. The crystal structure of the Orf virus NZ2 variant of VEGF-E has been solved by X-ray crystallography (PDB code: 2GNN, Figure 1) [16], as well as the co-crystal structure of VEGF-E in complex with VEGFR-2 (PDB code: 3V6B, Figure 10) [72]. These two structures show that the core regions of VEGF-E are very similar to the core regions of VEGF-A.

VEGF-Fs are isolated from snake venoms, for example, from the snake *Trimeresurus flavoviridis*, and they display a variety of structures and functions among different species [161]. The crystal structures of two VEGF-Fs vammin and VR-1 have been solved (PDB code: 1WQ8 and 1WQ9, Figure 1), and they exhibit significant differences from other known VEGFs, which may explain their high specificity for the VEGFR-2 [15]. A recent study indicated that vammin could induce VEGFR-2/NRP-1 related signaling more efficiently than VEGF-A [162]. VEGF-F has the potential to bind heparin, but it has almost lost the C-terminal heparin-binding domain compared with other heparin-binding VEGFs. Based on this observation, it was found that the C-terminal heparin-binding peptide of VEGF-F is able to inhibit VEGF-A-regulated endothelial cell proliferation [163,164]. As far as we know, no co-crystal structure of a ligand in complex with any VEGF-F has been reported.

## 6. Conclusions

From this overview of ligands of the VEGF family of growth factors, VEGF-A has been much more studied than the other members have. VEGF-A ligands comprise a variety of receptor fragments, aptamers, antibodies, mini proteins, and peptides. All the available ligand binding sites partially covered the receptor-binding interfaces and consequently acted as competitive inhibitors of VEGFRs. Targeting the receptor-binding epitopes of VEGF-A has been a successful approach and has been achieved through several conserved non-covalent interactions shared across the ligands. The smallest efficient ligands are peptides of 10–15 amino-acid length. Smaller peptides or small molecules may not be able to bind efficiently to the large flat hydrophobic surface of VEGFR binding sites. However, it seems that only a very small number of published studies have been aimed at that purpose. Future studies in this direction may be more successful, as our knowledge of this growth factor improves and new computational and experimental methods are being developed. Therapeutic applications of VEGF-A targeting have been only mediated through macromolecules. Although successful, it has shown some limitations. The use of small molecules or peptides may improve the outcome of this targeting due to their potentially different administration modes and pharmacokinetic properties.

Apart from VEGF-A, the development of ligands of the other members of the VEGF family has been strikingly lacking. Only receptor fragments and antibodies have been described, except a peptide series targeting PlGF. Future development of new ligands with increased potency and specificity and varying pharmacokinetic properties is warranted. It may help decipher further the biological activities of the VEGF family members and reveal or harness potential therapeutic applications.

## Figures and Tables

**Table 1:** Structural and affinity data for VEGF ligands for which a co-structure with the growth factor has been published.

VEGF Member	Ligand	Bound Epitope(s) of VEGF ^a^	Affinity/Method	Reference/PDB Code
VEGF-A	VEGFR-1_D2_	Monomer one: - helix α1 (16–27) - loop 2 connecting β3 to β4 (61–66) - strand β7 (103–106) Monomer two: - strand β2 (46–48) - strands β5 and β6 with the connecting turn loop 3 (79–91)	IC_50_ = 1.4 nM/ELISA type assay with biotinylated VEGF_8-109_ IC_50_ = 3.0 nM (VEGF_8-109_) SPR competition assay [81]	1FLT [69] or 1QTY [61]
	VEGFR-2_D2-3_	Idem above (helix α1, loops 2 and 3) + loop 1, which interacts solely with D3	K_d_ = 170 nM (VEGF_165_)/ITC: unfavorable binding enthalpy K_d_ = 18 ± 5.2 nM (VEGF_165_)/ITC [82] K_d_ = 36.7 ± 5.9 nM (VEGF_165_)/SPR [82]	3V2A [72]
	VEGFR-1_D1-6_	Residues interacting with D2 Monomer one: - helix α1 (M17, F18, Y21, Q22, Y25) Monomer two: - strand β2 (I46, K48) - strand β4 (Q79, M81, I83) - strand β5 (Q89, I91) Residues interacting with D3 Monomer one: - E64, D63 in L2 Monomer two: - P40, I43, E44 in L1 - K84, P85 in L3	K_d_ = 47 nM (VEGFR-1_D1-3_)/ITC K_d_ = 1.7 nM (VEGFR-1_D1-7_)/ITC	5T89 [70]
	Neuropilin-1	- D143 and K147 in helix 3, Q150, residues 152–156, and C-terminal residues 162–165 (VEGF_165_-A numbering)	K_d_ = 3.0 ± 0.2 nM (VEGF_164_)/ELISA type assay with AP-VEGF_164_	4DEQ [77]
	Fab-12 (refer to Y0192 in the article)	Monomer one: - F17 and Y21 of α1 - Y45 and K48 of α1- β2 Monomer two: - Q79-M94 of β5-β6 (except P85)	IC_50_ = 4.7 nM/Fab-phage ELISA; K_d_ = 3.4 ± 0.9 nM (25 °C)/SPR [8] K_d_ = 13 ± 2.2 nM [37 °C, VEGF_109_]/BIAcore SPR; K_d_ = 21 ± 3.8 nM [37 °C, VEGF_165_]/SPR; IC_50_ = 9 nM (37 °C)/ELISA assay with Fab12-IgG and Biotin-VEGF_109_; K_d_ = 0.433 nM (25 °C)/radiolabeled VEGF binding assay using VEGF competition with [^125^I] VEGF for binding to Fab [83]	1BJ1 [79]
	Y0317-Fab	Same as Fab-12 (binding site centers on the 80′s loop of VEGF)	K_d_ ≤ 0.14 nM [25 °C, VEGF_109_]/SPR; K_d_ = 0.11 ± 0.02 nM [37 °C, VEGF_109_]/SPR; K_d_ = 0.14 ± 0.05 nM [37 °C, VEGF_165_]/SPR;IC_50_ = 1 nM/ELISA assay with Biotin-VEGF_109_ K_d_ = 0.02 nM (25 °C)/radiolabeled VEGF binding assay using VEGF competition with [^125^I] VEGF for binding to Fab [9]	1CZ8 [83]
	DutaFab	- helix α1, extending to β1 - residues from β3, β5 and β7 strands	IC_50_ = 34 pM/ELISA assay with VEGF_165_	6T9D [84]
	Dual dAb	Similar to VEGFR-1_D2_	Mammalian cell-derived hVEGF_165_: K_d_ = 3.27 pM; E. coli expressed hVEGF_165_: K_d_ = 3.14 pM/T200 SPR hVEGF-A_165_: EC_50_ = 32 ± 2.7 pM; hVEGF_121:_ EC_50_ = 127 ± 22.1 pM; mVEGF_164:_ EC_50_ = 36 ± 4.7 pM/Mesoscale discovery binding assay VEGFR-1 IC_50_ = 59 ± 11.8 pM;VEGFR-2 IC_50_ = 22 ± 2.7 pM/receptor binding assay by Mesoscale discovery	V_K_·dAb: 5FV1 V_H_ dAb: 5FV2 [85]
	G6-Fab	Monomer one: - F17, M18, Y21, Q22, Y25, D63 Monomer two: - I83, H86, Q89, I91	Anti-mVEGF IC_50_ = 0.6 nM/Fab-phage ELISA; K_d_ = 0.91 nM/SPR [86]	2FJG [87]
	B20-4-Fab	Monomer one: - F17, M18, D19, Y21, R23, Y25 Monomer two: - Q89	K_d_ = 12 nM	2FJH [87]
	YADS1-Fab	The structural epitopes for binding to YADS1-Fab and YADS2-Fab overlap with each other and also with the structural epitope for binding to VEGFR-1_D2_	For hVEGF K_d_ = 1.8 ± 0.3 nM For mVEGF K_d_ > 1000 nM/SPR	1TZH [88]
	YADS2-Fab		For hVEGF K_d_ = 10 ± 2 nM For mVEGF K_d_ = 5.0 ± 0.8 nM/SPR	1TZI [88]
	D1-Fab	The structural epitope overlaps with the structural epitope for VEGFR-1_D2_	K_d_ = 7.8 nM/BIAcore SPR	2QR0 [89]
	Peptide v108	Monomer one: - 89–95 of β6 - 79–82 of β5 - 38–42 of α2 - 48 of β2 Monomer two: F17	IC_50_ = 8.2 μM/ELISA biotinylated VEGF_8-109_ [90]	1VPP [90]
	Peptide v107	Monomer one: - F17-C26 - C61-L66 - E103-R105 Monomer two: - F47-S50 - I89-R82 - Q89	IC_50_ = 1 μM/ELISA type assay with biotin labeled-v107	1KAT [91]
	D-RFX001	Bind to the same region of VEGF-A that interacts with VEGFR-1_D2_; cover much of the contact surface that VEGF-A uses to interact with VEGFR-1_D2_; Surface area of the binding interface is 800 Å^2^	K_d_ = 85 ± 12 nM/SPR, in a ProteOn™ XPR36 Protein Interaction Array System	4GLN or 4GLS [92]
	D-RFX037	Identical to D-RFX001 Surface area of the binding interface is 1350 Å^2^	K_d_ = 6.43 ± 0.07 nM/SPR, in a ProteOn XPR36 Protein Interaction Array System	5HHD or 5HHC [93]
	Z-Domain	Overlaps with the VEGFR-1_D2_ binding interface	IC_50_ = 343 nM/phage ELISA K_d_ = 38 nM/Octet binding assay	3S1K [94]
	Mini-Z dimer	Overlaps with the VEGFR-1_D2_ binding interface	IC_50_ = 227 nM/phage ELISA K_d_ = 40 nM/Octet binding assay	3S1B [94]
	Alpha/beta	Overlaps with the VEGFR-1_D2_ binding interface	K_i_ = 0.11 μM/FP assay	4WPB [95]
VEGF-B	VEGFR-1_D2_	Monomer one: - N-terminal helix α1 (Q11, W17-I18, Y21-T22, T25-Q27) - loop 2 connecting β3 to β4 (P62-D63, G65-L66) - C-terminal residues (E102-P105) Monomer two: - β2 (V48) - loop3 connecting β5 and β6 (L81, I83, S88-L90)		2XAC [96]
	2H10-Fab	Monomer one: - N-terminal helix α1 (16–24) - loop connecting β3 (51–58) to β4 (66–69) Monomer two: - loop connecting β2 (46–48) and β3.	K_d_ = 113.7 pM (VEGF-B_10-108_)/SPR IC_50_ = 3.4 nM (VEGF-B_10-108_)/cell-based assay	2VWE [97]
PlGF	VEGFR-1_D2_	Monomer one: - N-terminal helix (24–33) - loop connecting strands B and C - C-terminal residues 110–114 Monomer two: - AB loop (54–56) - CD loop (87–99)	IC_50_ = 275 nM (PlGF_19-116_)/SPR competition assay	1RV6 [81]
VEGF-C	VEGFR-2_D2-3_	Monomer one: - N-terminal helix α1 (113–129) - loop L2 (167–171) Monomer two: - loop L1 (139–155) - loop L3 (188–196)	K_d_ = 16 ± 6.7 nM/ITC with a VEGF-C mutant C137A K_d_ = 18.2 ± 5.3 nM/SPR with a VEGF-C mutant C137A	2X1X or 2X1W [82]
	VEGFR-3_D1-2_	The overall complex architecture is very similar to that of previously reported VEGFR-1 and VEGFR-2 structures VEGF-C binding is limited to D2, with D1 protruding away from VEGF-C	K_d_ = 250 nM/ITC with VEGF-C mutant C137A	4BSK [98]
	Neuropilin-2 (with C-terminus of VEGF-C)	R164 and R165 (VEGF_165_-A numbering)	ELISA type assay with AP-VEGF-C differential scanning fluorimetry (DSF) thermal shift assay (no data about K_d_ or IC_50_ of VEGF-C C-terminus binding to Neuropilin-2)	4QDQ [99]

^a^ Numbering corresponding to the sequence alignment in Figure 2.

## Data Availability

Not applicable.
